# 

**Published:** 1987-08

**Authors:** 


### Contents

**65 Editorial**

**66 The AIDS Epidemic and its control**

John Seale

**69 The origin of AIDS**

J. M. A. Smith

**73 Adverse reaction to Sulphasalazine**

M. Hamilton, A. J. K. Williams, M. J. Hall

**74 Axillary aneurysm presenting as a brachial plexus palsy.**

M. I. Aldoori, W. E. G. Thomas, K. Jeyasingham.

**75 Ten years on**

Rebecca B. Dunn, Mary E. McGraw

**76 From our Correspondents**

**78, 80 and 81 Book Reviews.**

**79 From our Foreign Correspondents**

**81 Correspondence**

**82 Obituaries, Dr H. J. Orr-Ewing and Dr James Macrae**

**84 South West Orthopoedic Club**

Hereford Meeting, May 1986, abstracts of papers

